# Natural variation in macrophage polarization and function impact pneumocyte senescence and susceptibility to fibrosis

**DOI:** 10.18632/aging.204309

**Published:** 2022-09-28

**Authors:** Eun Joo Chung, Seokjoo Kwon, Uma Shankavaram, Ayla O. White, Shaoli Das, Deborah E. Citrin

**Affiliations:** 1 Radiation Oncology Branch, Center for Cancer Research, National Cancer Institute, National Institutes of Health, Bethesda, MD 20892, USA

**Keywords:** senescence, macrophage, alveolar epithelial cell Type II, strain

## Abstract

Radiation-induced pulmonary fibrosis (RIPF), a late adverse event of radiation therapy, is characterized by infiltration of inflammatory cells, progressive loss of alveolar structure, secondary to the loss of pneumocytes and accumulation of collagenous extracellular matrix, and senescence of alveolar stem cells. Differential susceptibility to lung injury from radiation and other toxic insults across mouse strains is well described but poorly understood. The accumulation of alternatively activated macrophages (M2) has previously been implicated in the progression of lung fibrosis. Using fibrosis prone strain (C57L), a fibrosis-resistant strain (C3H/HeN), and a strain with intermediate susceptibility (C57BL6/J), we demonstrate that the accumulation of M2 macrophages correlates with the manifestation of fibrosis. A comparison of primary macrophages derived from each strain identified phenotypic and functional differences, including differential expression of NADPH Oxidase 2 and production of superoxide in response to M2 polarization and activation. Further, the sensitivity of primary AECII to senescence after coculture with M2 macrophages was strain dependent and correlated to observations of sensitivity to fibrosis and senescence *in vivo*. Taken together, these data support that the relative susceptibility of different strains to RIPF is closely related to distinct senescence responses induced through pulmonary M2 macrophages after thoracic irradiation.

## INTRODUCTION

Radiation therapy is a commonly used curative treatment modality for cancer patients. Irradiation (IR) of tumors typically results in the exposure of surrounding normal tissue to radiation, resulting in late adverse effects, such as fibrosis. Increasing radiation dose, larger treatment volumes, and the presence of underlying genetic sensitivity syndromes may increase risk of these injuries. Regardless, predicting an individual patient’s risk of fibrosis remains challenging.

Mouse models have been extensively studied to understand the molecular events contributing to radiation lung injury. The chronology of injury and severity of lung injury from a variety of stimuli have long been known to vary by strain [1–7], however the molecular events and processes responsible for these observed differences remain uncertain. Understanding differential susceptibility to RIPF between mouse strains may provide hypotheses to test in patients with divergent fibrotic responses to similar radiotherapy regimens and eventually may lead to new therapeutic targets, to alleviate this injury.

Recent studies using fibrosis-prone mouse strains have suggested that exposure of lung to fibrosis evoking doses of radiation may lead to senescence in type II pneumocytes (AECII), a cell that functions as an alveolar stem cell after lung injury by repopulating both type I and II pneumocytes. Reduction of AECII senescence by treatments to prevent senescence or elimination of senescent cells by senolytic agents has been demonstrated to prevent or ameliorate lung fibrosis after radiation [8–10]. AECII are known to be in close contact with alveolar macrophages, and, in this fashion, to contribute to lung homeostasis [11].

Macrophages are a key component of the immune cell response to radiation in lung [8, 12, 13]. Type II cytokines that are increased in irradiated lung, such as IL-13, may induce tissue-resident macrophages in lung to polarize into pro-fibrotic alternatively activated (M2) macrophages. M2 macrophages have been implicated in fibrotic progression in lung and other organs that occurs as a result of varying injurious stimuli [13–17]. Prior studies have identified differences in the accumulation of macrophage subsets in different mouse strains after thoracic irradiation, although the functional consequences of these differences are unexplored [18]. Recently, studies in a fibrosis sensitive strain have demonstrated that accumulation of M2 macrophages may be blunted through inhibition of senescence and the senescence associated secretory phenotype (SASP) [13, 14]. The impact of strain variation in these processes and their contribution to fibrosis has not been explored.

We hypothesized that intrinsic differences in macrophage function across strains contribute to variation in susceptibility to radiation lung injury. To explore this hypothesis, we evaluated three strains with varying sensitivity to radiation lung fibrosis (C57L – prone, C57BL6/J – intermediate, C3H/HeN – resistant). Global transcriptomic profiling of irradiated lung identified distinct categories of differentially expressed genes in response to radiation across the three strains. In the fibrosis prone C57L strain, enrichment analysis identified ROS and NO production in phagocytes as a pathway of interest. Several genes in the category encode components of NOX2, a molecule that produces superoxide radicals, which was in turn expressed to the greatest degree in M2 macrophages in C57L mice and in the lungs of irradiated C57L mice. Studies in bone marrow derived macrophages identified differential degrees of response to the polarizing stimuli IL-13, in which the expression of M2 markers and superoxide production via NOX2 was greatest in BMDM derived from fibrosis prone C57L mice and least in BMDM from fibrosis resistant C3H/HeN mice. This difference in macrophage superoxide production correlated to the capacity of M2 macrophages to induce senescence in co-cultured AECII, and rates of senescence *in vivo*. This study demonstrates that mouse strains with varying sensitivity to fibrosis exhibit differential rates of senescence after the same stimuli and provides the first evidence that M2 macrophages participate in the late effects of radiation through induction of AECII senescence.

## MATERIALS AND METHODS

### Mice and irradiation

C57Bl6-J (Stock No: 000664), C57L (Stock No: 000668) and C3H/HeN female mice were obtained from the Jackson Laboratory (Bar Harbor, ME, USA) or the Division of Cancer Treatment, National Cancer Institute (Frederick, MD, USA). Female mice were selected to avoid sex-based differences confounding results, as one of the three selected strains has shown sex-based variation in response [6]. Further, female mice were used as macrophage infiltration [19], polarization [20, 21], and function [22] is known to vary by sex in mice. Ten-week-old mice were restrained in a custom Lucite jig with lead shielding that allowed for selective irradiation of the thorax (n>5 per condition). Five daily fractions of 6 Gy were delivered to the thorax with an X-RAD 320 (Precision X-Ray, North Branford, CT, USA) using 2.0 mm Al filtration (320 kv peak) at a dose rate of 1.9 Gy/min. The radiation regimen used has been shown to lead to lethal pulmonary fibrosis and has been used extensively in the study of lung injury in the context of senescence, macrophage infiltration and polarization, and mitigation of radiation injury [8, 14, 23–27]. Dosimetry was confirmed in phantoms using thermoluminescent dosimeters. At the indicated timepoints, lung was snap frozen and stored at -80° C, inflated with 10% neutral buffered formalin and paraffin embedded, or frozen in OCT compound for frozen sections.

### Bronchioalveolar lavage collection and analysis

Lungs from mice (n>5 per condition, 15 weeks after radiation) were pulsed with 1 ml ice cold 5 mM EDTA instilled via intratracheal catheter. The lavage was repeated and pooled washings were centrifuged at 300 × *g* for 10 minutes. The cell pellet was resuspended in 2% BSA. After blocking the Fc receptor with purified anti-mouse CD16/CD32 antibody (#101335, BioLegend, San Diego, CA, USA), cells were labeled with a fluorophore-conjugated antibodies against F4/80 (#123116, BioLegend). Labeled cells were fixed with 2% PFA, permeabilized using a Permeabilization kit (FIX and PERM, Thermo Fisher, Waltham, MA, USA), and then labeled with an anti-Arginase 1 antibody (#89872, Cell Signaling Technology, Beverly, MA, USA) followed by an appropriate secondary antibody conjugated with a fluorophore (Thermo Fisher). At least 100,000 events were acquired for each sample with a CytoFlex (Beckman Coulter, Brea, CA, USA) and data were analyzed with FlowJo software (Tree Star, Inc., Ashland, OR, USA).

### Histopathology and histochemistry

For immunohistochemical staining, 5 μm thick lung sections were deparaffinized in xylene and rehydrated through a graded alcohol series. Antigen retrieval was performed in citrate buffer (pH 6.0, Vector Labs, Burlingame, CA, USA) in a pressure cooker. Endogenous peroxidase was quenched with 0.3% H_2_O_2_ for 10 minutes. Sections were blocked with 2.5% normal horse serum for 1 hour and incubated with primary antibodies at room temperature for 2 hours. Primary immunoreactivity was detected with a polymeric peroxidase-conjugated secondary antibody (ImmPress, Vector Labs) and visualized by 3,3’-diaminobenzidine histochemistry (ImmPACT, Vector) in murine lung sections. Antibodies against F4/80 (70076), Arginase-1(93668), and CD86 (19589) were purchased from Cell Signaling Technology (Beverly, MA, USA). The number of each type of macrophage was counted on five 20x fields per mouse (n>3 mice per condition) in whole lung or adjacent fibrotic regions (within 400 μm). An antibody against NOX2 (ab80897), F4/80 (ab6640), and CD206 (24595) were purchased from Abcam (Cambridge, MA, USA) and Cell Signaling Technology (Danvers, MA, USA). Sections were counterstained with Harris Hematoxylin (Avantik, Pine Brook, NJ, USA), dehydrated, and mounted with Permount (Thermo Fisher).

Senescence-associated β-galactosidase (β-gal) activity was assessed with a commercially available assay (Abcam, Cambridge, MA, USA). The number of senescent cells was counted on five 20x fields per mouse (n>3 mice per condition) and expressed as a percent of total AECII cells, which were visualized with anti- anti-prosurfactant protein C antibody (NBP1-87201, Novus Biologicals, Centennial, CO, USA) and a compatible secondary antibody conjugated to Alexa Flour 594 (A-21207, Thermo Fisher, Waltham, MA, USA). Labeled sections were counterstained with 4’,6-diamidino-2-phenylindole (DAPI, D9542, Sigma Aldrich) and mounted with Prolong Gold Anti-Fade Reagent (P36930, Thermo Fisher). Stained tissues were examined on a Leica DMRXA microscope (Wetzlar, Germany) equipped with a QImaging Micropublisher Camera (Surrey, BC, Canada).

For histologic evaluation of fibrosis, formalin-fixed paraffin-embedded lung sections were deparaffinized in xylene, rehydrated, and stained with Masson’s trichrome stain kit (HT15, Sigma-Aldrich) and Weigert’s iron hematoxylin solution (HT107, Sigma-Aldrich) according to manufacturer instructions. Stained sections were scanned using an Aperio AT2 digital scanner (Leica Biosystems Inc., Buffalo Grove, IL, USA).

### Hydroxyproline assay

Hydroxyproline content was measured using a Hydroxyproline assay kit (MAK008, Sigma-Aldrich) according to the manufacturer’s protocol. Hydroxyproline amount was calculated based on total lung weight and expressed as micrograms per lung.

### NanoString assays

Total RNA was extracted from primary macrophages derived from bone marrow using the RNeasy Plus mini kit (Qiagen, Valencia, CA, USA). Isolated RNA was quantified using the DS-11 spectrophotometer (DeNovix, Wilmington, DE, USA). A Customized Code Set for the NanoString assay contained probes against target genes related to senescence, inflammation, and fibrosis (Supplementary Table 1). Probes and 500 ng total RNA from each sample were hybridized overnight at 65° C according to the manufacturer’s protocol. A NanoString nCounter Digital Analyzer (NanoString Technologies, Seattle, WA, USA) was used to count the digital barcodes representing the number of transcripts. The raw expression data were normalized using nSolver Analysis software. A normalization factor was calculated by obtaining the geometric mean of the positive controls used for each sample and applied to the raw counts of the nCounter output data to eliminate variability that was unrelated to the samples. The resulting data were normalized again with the geometric mean of the housekeeping genes.

### Quantitative PCR

Total RNA from tissue or cells was extracted with Trizol reagent (Life Technologies) and purified with the RNeasy plus system (Qiagen), and reverse-transcribed using QuantiTect reverse transcription kit (Qiagen). Quantitative PCR (qPCR) was performed on an ABI 7500 system (Applied Biosystems) using predesigned primer and probe sets (Supplementary Table 2) for Taqman gene expression assays (Life Technologies, Grand Island, NY, USA). The change of target mRNA expression was normalized to endogenous actin.

### Cell isolation

Primary AECII cells were isolated from female C57BL6/J, C57L and C3H/HeN mice, aged 10 weeks, as previously described [27]. Briefly, mice were anesthetized 10 minutes after intraperitoneal injection of Heparin Sodium (100 USP unit/mouse, Fresenius Kabi USA. LLC. Lake Zurich, IL, USA). Lungs were perfused with 10 ml of HBSS (Thermo Fisher) containing 30 mM HEPES (Sigma-Aldrich), filled with 1 mL enzyme cocktail in HBSS containing 30 mM HEPES (Elastase 3 u/ml, 0.01% DNase I and 0.2 % Collagenase, Sigma-Aldrich), and incubated in 5 ml of enzyme cocktail per lung at 37° C for 30 minutes. The digested tissue was carefully teased from the airways using a tissue scissor and a scalpel, transferred into a conical tube, and gently swirled in a water bath (37° C) for 5 to 10 minutes. The resulting suspension was successively filtered through 100 mm and 40 mm Falcon cell strainers, then centrifuged at 130 x g for 8 min at 4° C and resuspended in HBSS. The crude single cell suspension was applied to discontinuous layers of Ficoll density gradients (Histopaque-1077, 1083, Sigma-Aldrich). After centrifugation at 400 x g for 25 min (4° C), pneumocytes were collected from the layer of density 1.077 ~ 1.083, washed twice with HBSS (400 x g for 10 min, and 130 x g for 8 min at 4° C), and then resuspended with DMEM media containing 10 % FBS and 1% antibiotics. Isolated cells were used for *in vitro* senescence assays or for RNA isolation as described above.

### Bone marrow derived macrophage cultures

Bone marrow monocytes were enriched from each strain mice femur and tibia, and pre-differentiated into macrophages (M0) by 6-day culture in RPMI/10% FCS supplemented with 20 ng/ml of M-CSF. M0 macrophages were cultured with each stimulus (1 ng/ml LPS, 10 ng/ml IL13 or PBS). For the induction of AECII senescence, the polarized macrophages were co-cultured with AECII isolated from mice of each strain.

### Western blotting

Macrophage extracts were prepared using Cell lysis buffer (Cell Signaling Technology) containing Halt™ Protease and Phosphatase Inhibitor Cocktail (Thermo Fisher Scientific, Waltham, MA, USA) and PMSF (Sigma-Aldrich), followed by measurement of protein concentrations by the Bradford method (Bio-Rad, Hercules, CA, USA). Equal amounts of protein were subjected to western blot analysis, which were probed with the following primary antibodies: NOX1 (ab131088), NOX2 (ab80897, Abcam, Cambridge, UK), NCF1 (PA5-104250), NCF2 (PA5-37323), NCF4 (PA5-102575, Thermo Fisher Scientific) and Actin (MAB1501, Millipore Sigma, Burlington, MA, USA). ImageJ software (NIH, Bethesda, MD, USA) was used to evaluate the relative expression of each molecule normalized to actin.

### Co-culture with AECII and polarized macrophages

To evaluate the induction of AECII senescence by macrophages, AECII cells were co-cultured with polarized BMDM using Transwell inserts (Nunc-140652, 0.4 μm, Thermo Fisher). Briefly, 5x10^5^ macrophages polarized with vehicle or IL13 were seeded on the bottom of well (Nunc 12-well culture plate), 4x10^4^ AECII into the insert, and co-cultured with 2 ml DMEM media containing 10% FBS and 1% antibiotics for 72 hours. AECII on membrane was washed with PBS twice, applied to β-galactosidase activity assay (Abcam). AECII was visualized with anti-prosurfactant protein C antibody (Abcam, Cambridge, MA, USA) and a secondary antibody conjugated to Alexa Flour 594 (Thermo Fisher). AECII on membrane was placed on glass slide (VWR, Radnor, PA, USA), and mounted with ProLong antifade reagent containing DAPI (Thermo Fisher). The number of senescent AECII was counted on five 20× fields per mouse (n ≥ 5).

### Superoxide production

The production of superoxide anion in cell cultures was measured using the Superoxide Anion Assay kit (CS1000, Sigma-Aldrich) in polarized macrophages according to manufacturer’s instruction. Briefly, polarized macrophages (5x10^4^ cells per well) resuspended with 100 μl Assay Medium (A5980) were added into 100 μl assay buffer (A5980) containing 5 μl luminol (L5043), 5 μl enhancer (E4281) and 1 μl PMA (final concentration 100 ng/ml, p1585). Superoxide generation was measured by monitoring chemiluminescence (relative light units, RLU) using a Synergy H1 plate reader (BioTek, Winooski, VT, USA).

In tissues, superoxide anion production was evaluated by dihydroethidium (DHE) staining in murine lung. Frozen lung sections (5 μm thickness) were rinsed with cold PBS twice to remove OCT compound and incubated with PBS containing 5 μM dihydroethidium (DHE; D-1168, Thermo Fisher) and 1 μg/ml DAPI (Sigma-Aldrich, D9564) for 5 minutes at room temperature in the dark. The sections were rinsed 2 times with PBS, mounted with prolong gold antifade mounting reagent, and examined the fluorescence immediately using a Leica DMRXA microscope (Wetzlar, Germany) equipped with a QImaging Micropublisher Camera (Surrey, BC, Canada). The percent area of DHE positive cells was measured from 40 different regions in each group of lung tissue using ImageJ Software (National Institutes of Health, Bethesda, MD, USA; open source: https://imagej.nih.gov/ij/docs/index.html).

### RNA-sequencing

Frozen lung tissue (20-30 mg) was mechanically homogenized in TRIzol (Sigma-Aldrich) and incubated for 5 min at RT to allow complete dissociation of nucleoprotein complexes. 200 μL of chloroform (Sigma-Aldrich) was mixed with 1 mL homogenate, incubated for 5 minutes, and centrifuged. The resulting aqueous phase was transferred to gDNA eliminator column, and RNA was isolated from the flow through using RNeasy Plus Mini isolation kit (QIAGEN, Cat # 74134). RNA integrity was assessed using the Agilent Bioanalyzer (Agilent Technologies, Santa Clara, CA, USA), and high quality of RNA (RIN ≥ 9) was used for further assay.

RNA Sequencing was performed using Illumina platform. Briefly, the ribosomal RNA (rRNA) was removed using biotinylated, target-specific oligonucleotides conjugated with Ribo-Zero rRNA removal beads. The cleaved RNA pieces after fragmentation were copied into the first strand cDNA using reverse transcriptase and random primers, followed by the second strand cDNA synthesis using DNA Polymerase I and RNase H. The resulting double-strand cDNA was used as the input to a standard Illumina library prep with end-repair, adapter ligation and PCR amplification being performed to prepare a sequencing ready library. The final purified product was quantitated by qPCR before cluster generation and sequencing. Reads of the samples with a base call >Q30 were retained. The resulting reads were trimmed for adaptors and low-quality bases using Trimmomatic software before alignment to Ensembl release 70 Mouse mm10 reference genome using TopHat_v2.0.8 software. Given the aligned sequencing reads and a list of genomic features, counts of mapped reads for each gene were calculated using HTSeq [28]. Overall, 77% of reads were mapped to unique alignments. CCBR Pipeliner (https://github.com/CCBR/Pipeliner) was used for processing of raw sequences with default settings. RSEM [29] was then used for gene-level expression quantification, and EdgeR [30] was used for normalization and to correct the reads for sequencing depth. Differentially expressed gene identification between control and treated samples was performed using Limma package [31] with multiple comparison correction by Benjamini Hochberg method. The significant genes were identified using threshold of adjusted p value < 0.05. Results were summarized by Venn diagrams and Heatmap computed with Euclidean distance and Pearson correlation distance. Ingenuity pathway analysis was used for functional annotation of genes based on EdgeR normalized counts. Single sample gene set variation analysis (GSVA) was done with a signature gene set for 25 immune cell types in mouse, collected from the ImmuCC model. Immune gene sets differentially enriched between the control and treated samples was identified using Limma package [32, 33].

### Statistical analyses

All data are presented as mean ± SD unless otherwise noted. For collagen content, histochemistry, immunohistochemistry, PCR, and tissue-based analyses, a sample size of > 5 mice per condition and time point was selected. In prior studies, including >5 samples was sufficient to resolve significant differences in these measures between irradiated and unirradiated mice at the 15-16 week time point, while providing information about variability between similarly treated mice. Statistical analyses were performed using either Mann-Whitney test (non-parametric statistical test) for comparisons between two conditions or ANOVA with Tukey’s correction for multiple comparisons. A p value of ≤0.05 was considered significant for comparisons. All studies in tissues were conducted in triplicate unless otherwise noted.

## RESULTS

### Susceptibility to radiation-induced lung injury and premature AECII senescence varies by mouse strain

**Figure 1:**
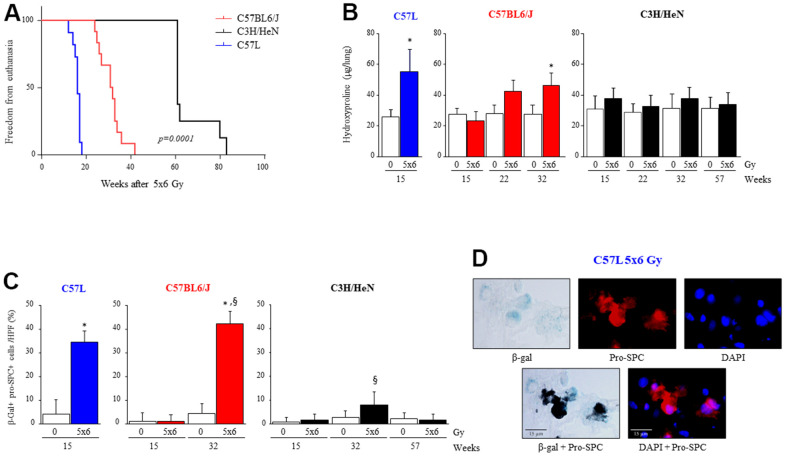
**Varying susceptibility to radiation induced pulmonary fibrosis and pneumocyte senescence among three strains of mice.** C57L, C57BL6/J, and C3H/HeN mice were exposed to 5 daily fractions of 6 Gy (5x6 Gy) of thoracic irradiation. At 15, 22, 32 and 57 weeks after irradiation, lung tissue was collected (n=5 mice per timepoint and condition). (**A**) Kaplan–Meier plot of freedom from euthanasia of irradiated mice (n≥10 mice per group) with comparison of curves using log rank test. (**B**) Hydroxyproline content was assessed in lung tissue at the indicated time point (in weeks) after irradiation. (**C**) Senescence associated-β-Galactosidase activity was assessed in lung samples collected at the indicated time points after irradiation, followed by immunocytochemical localization of pro-surfactant C. The percent of senescent AECII was scored. Columns: mean, error bars: +SD, ^*^p<0.05 for comparison to 0 Gy for the corresponding strain and timepoint by ANOVA with Tukey’s correction. ^§^p<0.05 for comparison to C57L lungs exposed to 5x6 Gy by ANOVA with Tukey’s correction. (**D**) Representative images of costaining of tissue sections for senescence associated-β-Galactosidase activity and pro-surfactant C in the C57L strain.

To investigate the differences in lethal lung injury and pulmonary fibrosis after thoracic irradiation, ten-week old female mice of each strain (C57BL6/J, C57L or C3H/HeN) were exposed to 5 daily fractions of 6 Gy thoracic irradiation. Mice were followed for evidence of pulmonary morbidity, at which point mice were euthanized. The survival proportion after thoracic radiation was estimated using the Kaplan-Meier method (Figure 1A). The median survival time after thoracic irradiation was 15 weeks in C57L, 27 weeks in C57BL6/J and 80 weeks in C3H/HeN groups (p=0.0001). Pulmonary hydroxyproline content was elevated in irradiated C57L mouse lungs as early as 15 weeks after IR (0 Gy: 25.6 ± 4.7, 5x6 Gy: 55.3 ± 14.8 μg per lung, p=.0017), whereas a significant increase in hydroxyproline content after IR was not observed until 32 weeks in C57BL6/J mice (0 Gy: 27.7 ± 6.0, 5x6 Gy: 46.3 ± 7.9 μg per lung, p=.0065). Hydroxyproline content was not significantly different in irradiated and unirradiated C3H/HeN mice as late as 57 weeks after IR. Histologic assessment of fibrotic progression with Masson’s Trichrome staining (Supplementary Figure 1) was concordant with the hydroxyproline assay (Figure 1B), demonstrating relative sensitivity to fibrosis in the C57L strain, intermediate sensitivity to fibrosis in the C57BL6/J strain, and relative resistance to fibrosis in the C3H/HeN strain.

Premature senescence is a known consequence of oxidative stress [34–36], and premature senescence of AECII as a result of superoxide production is considered a key contributor to pulmonary fibrosis [13, 14, 27]. To investigate if the accumulation of senescent AECII after IR varied by mouse strain, β-galactosidase activity was assessed in lung tissue sections co-stained with immunohistochemistry for pro-surfactant-C (pro-SPC) at multiple timepoints after IR (Figure 1C, 1D). Accumulation of senescent AECII occurred by 15 weeks after thoracic IR in the lungs of fibrosis sensitive C57L mice (0 Gy: 4.3 ± 6.1, 5x6 Gy: 34.6 ± 4.6 % of total AECII per HPF, p<.0001). Senescent AECII accumulation in C57BL6/J lungs was not evident at 15 weeks after IR, but by 32 weeks a marked increase in senescent AECII was observed (0 Gy: 4.5 ± 4.1, 5x6 Gy: 42.3 ± 5.2 % of total AECII per HPF, p<.0001) (Figure 1C). Accumulation of senescent AECII was minimal in C3H/HeN mouse lungs as late as 57 weeks after IR. Thus, the pattern of senescent AECII accumulation after IR observed correlated with the degree of fibrosis across strains.

### RNA sequencing identifies strain specific responses to IR in lung

**Figure 2:**
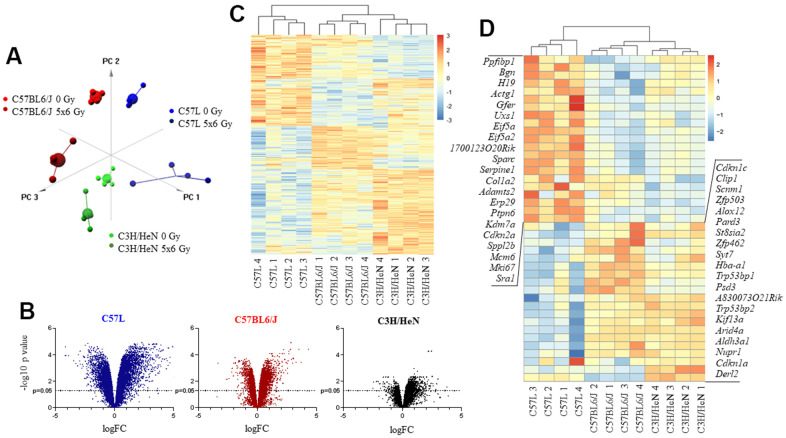
**Impact of thoracic irradiation on gene expression in lung tissue from three mouse strains with varying susceptibility to fibrosis.** Mice were exposed to 5x6 Gy thoracic IR or no IR (0Gy). Samples of lung tissue (n=4 per dose) were collected at 15 weeks after IR, RNA was isolated, and further evaluated with RNA sequencing. (**A**) Principal component analysis of differentially expressed genes for all evaluated groups (n=4 per condition). (**B**) Volcano plot of the ratio of gene expression (irradiated: unirradiated) and *p* value for each observed gene. (**C**) Unsupervised hierarchical clustering of evaluated samples based on different expression of genes after irradiation. Irradiated samples were evaluated relative to a paired unirradiated sample of the same strain, thus expression depicted is the ratio (irradiated: unirradiated). (**D**) Hierarchical clustering of samples based on different expression of senescence and aging genes after irradiation. Irradiated samples were evaluated relative to a paired unirradiated sample of the same strain, thus expression depicted is the ratio (irradiated: unirradiated).

To provide insights into the strain specific differences in lung injury, and to explore potential contributors to the observed differences in senescence, gene expression was evaluated with whole-transcriptome sequencing in lung tissues collected from each strain at 15 weeks after IR (0 Gy or 5x6 Gy, n=4 per condition). As an initial assessment of the patterns of gene expression in the three strains, principal component analysis (PCA) was performed. PCA indicated consistent expression patterns among the replicate samples for each condition with distinct expression profiles generated for each strain. Moreover, there were clear differences between the expression profiles obtained from irradiated versus unirradiated lung within and between strains. Thus, these studies indicate that gene expression in lung is distinct in the three strains in the presence or absence of radiation (Figure 2A).

The ratio of expression between radiated and unirradiated samples within and across each strain was then studied further. When comparing across the three strains, the greatest number of differentially expressed genes in response to IR at 15 weeks was observed in the C57L strain (n=3576), an intermediate number in C57BL6/J (n=1709), and the least in C3H/HeN (n=429). These data suggest that C57L mouse lungs are relatively hyperresponsive to radiation in terms of gene expression among the three strains and C3H/HeN mouse lungs are relatively hyporesponsive at the 15 week time point (Figure 2B). Unsupervised hierarchical clustering of genes that were differentially expressed after IR resulted in clear segregation by strain (Figure 2C). Prior work has demonstrated that a senescence and aging gene set is enriched in irradiated murine lung [27]. To determine if the patterns of senescent gene expression varied between strains, expression data were again subjected to hierarchical clustering using the 41 genes from AGEMAP (lung), mSS, and DASS [37, 38] that were differentially expressed across the three strains in response to radiation. This analysis resulted in three major clusters that were segregated by strain, confirming a strain specific response to radiation (Figure 2D).

**Figure 3:**
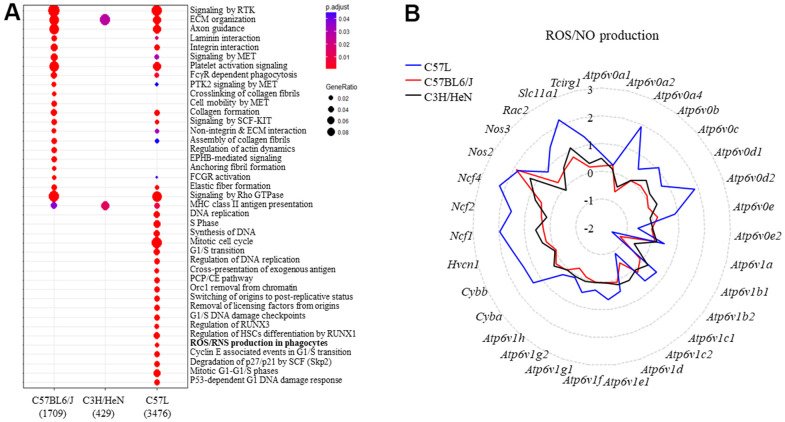
**Identifying the genes belonging to ROS and NO production and the types of inflammatory cells in mouse lungs after thoracic radiation.** (**A**) Reactome enrichment analysis of differentially expressed genes in the lungs of each strain (irradiated: unirradiated) at 15 weeks after irradiation. (**B**) The expression of genes in the ROS and NO production pathway were compared between the three strains (irradiated: unirradiated). The y-axis corresponds to the mean fold-change of each gene comparing irradiated to unirradiated within a strain (at 15 weeks after irradiation).

Enrichment analysis of differentially expressed genes in mouse lung after IR identified both overlapping processes and distinct pathways between the three strains (Figure 3A). Because the C57L strain demonstrates enhanced sensitivity to fibrosis, pathways enriched in this strain after IR were explored further. A number of DNA damage, cell cycle, and matrix related pathways were enriched in the C57L strain. In addition, the “ROS and NO production in phagocytes” pathway was enriched in C57L mouse lungs relative to the two other strains. This pathway is notable given that chronic oxidative stress is known to contribute to radiation lung injury through a variety of processes, including senescence [39, 40].

To further explore this finding, the ratio of the expression of genes (radiated to unirradiated) included in the Reactome pathway of “ROS and NO production in phagocytes” were compared between the three strains (Figure 3B). Several genes (*Ncf1, Ncf2, Ncf4, Cybb, Cyba)* from this pathway were differentially expressed only in the lungs of C57L mice after IR. Collectively, these transcripts encode components of NADPH oxidase 2 (NOX2), which is a major source of cellular reactive oxygen species in phagocytes and is essential for macrophage activation and polarization towards the M2 subtype [41]. The increased expression of components of NOX2 suggested a shift in the inflammatory landscape in irradiated C57L lung tissue relative to the other strains.

### Macrophage accumulation and phenotype in irradiated lung varies by strain

The accumulation of alternatively activated macrophages in lung is associated with the progression of pulmonary fibrosis [8, 13, 14]. To determine if the observed differences in susceptibility to fibrosis and senescence were associated with differences in macrophage accumulation across strains, mRNA for *Adgre1* (F4/80), *Cd86* and *Arg1* in lung tissue was evaluated in lung tissue from the three strains at 15 weeks after IR (Supplementary Figure 2A). The expression of *Adrege1* was increased substantially in C57L lung tissue, as was the expression of *Cd86* and *Arg1*. In contrast, the increase in expression of all three macrophage markers was significantly less in the lungs of irradiated C57BL6/J and C3H/HeN mice compared to irradiated C57L mice.

To further explore macrophage subsets in the different strains of mice after irradiation, the expression of macrophage polarization markers *Arg1*, *Cd163*, *Cd206*, *Il10*, *Tgfb1*, *Tlr1*, *Tlr8*, *Nos2*, and *Cd68* was evaluated in bronchioalveolar lavage cells from the three strains collected at 15 weeks after irradiation (Supplementary Figure 3A). Classical M1 markers were only slightly increased (in the case of *Cd68*) or not significantly impacted (in the case of *Nos2*) after irradiation in all three strains. Both *Arg1* and *Cd206* are considered markers for M2a and M2c sub-phenotypes, and both were increased after irradiation, most notably in C57L mice. In contrast, *Cd163* expression was not detectable in these cells (a marker for M2a and M2c). The M2c markers *tgfb*, *tlr8*, and *trl1* were only slightly altered after irradiation. Using flow cytometry, it was determined that Arginase-1 expressing cells were largely F4/80+ in bronchioalveolar lavage cells (Supplementary Figure 3B). Thus, both Arginase-1 and CD206 were considered to be representative of changes occurring in alveolar macrophages after irradiation *in vivo*, but further subclassification into M2a or M2c was not possible.

**Figure 4:**
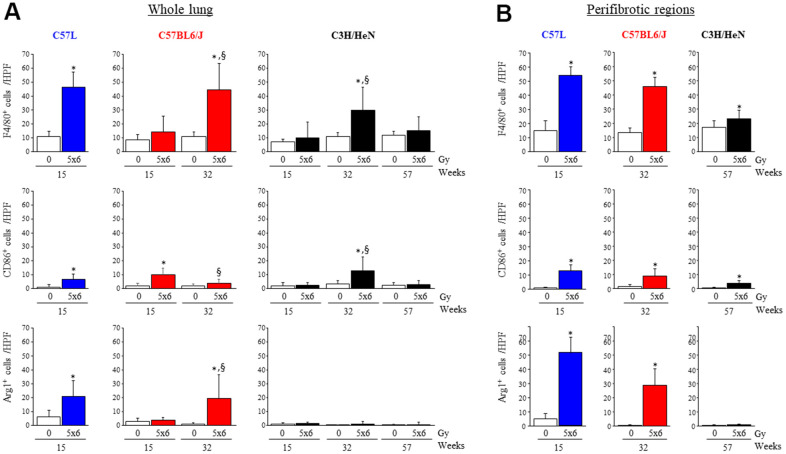
**Accumulation of macrophages in irradiated lungs from three strains of mice.** Immunohistochemical assays for (F4/80+), M1 macrophages (CD86+), and M2 macrophages (Agr-1+) were performed on lung tissue sections collected at the indicated timepoints after thoracic irradiation (5 x 6Gy). The numbers of cells were scored in whole lung (**A**) or within perifibrotic regions (within 400 μm of fibrotic regions) (**B**) in n=5 mice per time point and condition. Columns: mean, error bars: + SD, ^*^p<0.05 for comparison to the corresponding 0 Gy by ANOVA with Tukey’s correction. ^§^p<0.05 for comparison to lungs exposed to 5x6 Gy at 15 weeks by ANOVA with Tukey’s correction HPF: high power field (20X), IR: irradiation.

To confirm these findings and provide spatial context, immunohistochemical assays were performed using pan macrophage (F4/80), M1 (CD86) and M2 (Arg-1, CD206) markers (Figure 4A and Supplementary Figure 3C, 3D). F4/80+ macrophages accumulated in C57L mouse lungs by 15 weeks after IR (0 Gy: 10.7 ± 3.6, 5x6 Gy: 46.3 ± 10.9 cells/HPF), but not until 32 weeks in C57BL6/J (0 Gy: 10.5 ± 3.3, 5x6 Gy: 44.6 ± 18.8 cells/HPF) and C3H/HeN (0 Gy: 10.9 ± 2.7, 5x6 Gy: 29.5 ± 16.9 cells/HPF). Prior work has described differences in macrophage subpopulations in some strains of mice after irradiation [18]. Both M1 (CD86+) and M2 (Agr-1+) polarized macrophages accumulated in fibrosis sensitive C57L mouse lungs by 15 weeks after IR (M1- 0 Gy: 1.0 ± 1.5, 5x6 Gy: 6.4 ± 4.1, M2- 0 Gy: 5.9 ± 4.7, 5x6 Gy: 20.7 ± 11.5 cells/HPF). In the C57BL6/J strain that exhibits intermediate fibrosis sensitivity, the accumulation of M1 macrophages was significant in irradiated lung at 15 weeks, before the presence of fibrotic foci (0 Gy: 1.9 ± 1.9, 5x6 Gy: 9.6 ± 5.0 cells/HPF at 15 weeks after IR). By 32 weeks, M2 macrophage accumulation was also evident in irradiated C57BL6/J lungs (0 Gy: 0.8 ± 0.9, 5x6 Gy: 19.3 ± 16.9 cells/HPF at 32 weeks after IR). A similar pattern was observed with CD206 (Supplementary Figure 3C, 3D).

When considering macrophage distribution in peri-fibrotic areas, M2 macrophages accumulated primarily in or adjacent to fibrotic foci in irradiated C57L and C57BL6/J lungs, whereas M1 macrophages were distributed more evenly throughout the lung (Figure 4B and Supplementary Figure 2B). In the rare fibrotic foci in C3H/HeN lungs at 32 weeks, macrophages were mostly M1 type (0 Gy: 3.2 ± 2.3, 5x6 Gy: 12.5 ± 10.0 cells/HPF) rather than M2 type (0 Gy: 0.1 ± 0.2, 5x6 Gy: 0.9 ± 1.7 cells/HPF).

### Macrophages demonstrate strain dependent responses to polarizing stimuli

**Figure 5:**
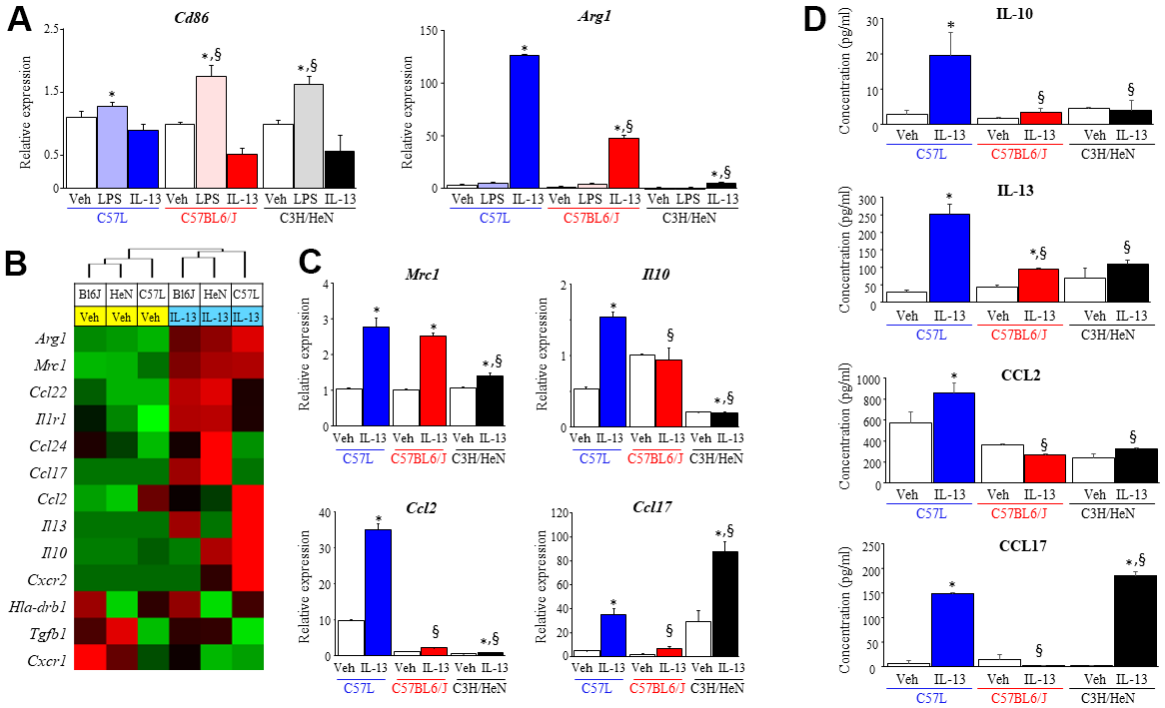
**Characterization of macrophage phenotype across mouse strains after exposure to polarizing stimuli.** Bone marrow derived macrophages from each strain were polarized with vehicle (PBS), LPS (1 ng/ml) or IL-13 (10 ng/ml). After 3 days of exposure to each stimulus, total RNA was isolated for further assays. (**A**) Polarization in response to LPS (M1) or IL-13 (M2) was evaluated by assessing the level of *Cd86* and *Arg1* mRNA with quantitative PCR (QPCR) normalized to β-actin mRNA. (**B**) The expression of genes related to M2 polarization was evaluated in macrophages treated with vehicle or IL-13 using the NanoString nCounter Gene Expression Assay and a custom code set. Unsupervised hierarchical clustering of M2 related genes was performed (left panel). (**C**) mRNA expression of *Mrc1*, *Il10*, *Ccl2* and *Ccl17* in polarized macrophages was confirmed by QPCR. (**D**) The concentrations of IL-10, IL-13, CCL2 and CCL17 in supernatants collected from polarized macrophages were determined with ELISA. Veh: PBS, BL6J: C57BL6/J, HeN: C3H/HeN. Columns: mean, error bars: +SD, ^*^p<0.05 for comparison to the corresponding macrophages treated with vehicle. ^§^p<0.05 for comparison to C57L macrophages exposed to IL-13 by ANOVA with Tukey’s correction.

As the accumulation of macrophage subsets in irradiated lung varied both temporally and quantitatively by strain, we hypothesized that the response of M0 macrophages to polarizing stimuli varied by strain. Bone marrow-derived macrophages (BMDM) were prepared from untreated donor mice of each strain and treated *in vitro* with polarizing stimuli (LPS to polarize M0 toward M1; IL-13 to polarize M0 toward M2) for 3 days (Figure 5). mRNA levels of the M1 marker, *Cd86*, were significantly increased after LPS treatment in all three strains (C57L: 1.3 ± 0.05, C57BL6/J: 1.7 ± 0.18, C3H/HeN: 1.6 ± 0.13 fold), however, BMDM derived from C57L mice exhibited reduced sensitivity to LPS compared to C57BL6/J and C3H/HeN (Figure 5A). In contrast, the expression of the M2 marker, *Arg1*, was significantly increased in all three strains after IL-13 stimulation (C57L: 126.3 ± 1.1, C57BL6/J: 47.9 ± 2.1, C3H/HeN: 5.1 ± 0.2 fold), but the increase of *Arg1* mRNA after IL-13 treatment was highest in C57L BMDM, moderate in C57BL6/J, and low in C3H/HeN.

IL-13 has been shown to play a key role in lung fibrosis from several stimuli, including radiation [8]. To further explore the differences in macrophage response to IL-13 across the three strains, the expression level of the genes associated with M2 macrophage polarization were assessed by NanoString assay and confirmed by quantitative PCR (Figure 5B and Supplementary Figure 4). The expression of *Arg1* and *Mrc1* was increased significantly among BMDM of all three strains in response to IL-13. However, for the other genes evaluated, BMDM from each of the three strains responded notably differently to IL-13 stimulation. BMDM derived from C3H/HeN mice exhibited increased expression of *Ccl17* and *Ccl24* mRNA relative to the two other strains, whereas the expression of *IL10*, *IL13*, and *Ccl2* were highest in BMDM derived from C57L mice. The protein levels of these cytokines (IL10, IL13, CCL2 and CCL17) were evaluated in BMDM from each strain in culture supernatants collected after polarization with IL-13 (Figure 5C). The protein concentration of these cytokines correlated with the pattern observed in the NanoString assay. The varied expression of M2 phenotype-related molecules across the three strains led us to question whether strain specific macrophage responses could contribute to the differential susceptibilities of each strain to RIPF.

### M2 macrophages induce AECII senescence via NOX2-derived superoxide production in a strain dependent manner

**Figure 6:**
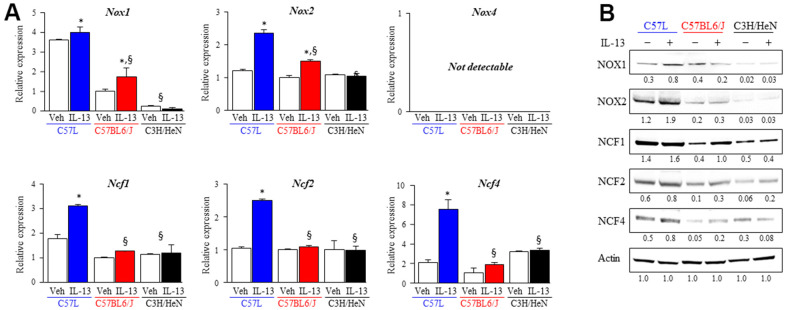
**Increased expression of components of NOX complexes and ROS production in M2 macrophages from fibrosis sensitive strains.** The expression of ROS producing enzymes (NOX1, NOX2, NOX4) and subunits (NCF1, NCF2, NCF4) of the NOX2 complex was assessed in M2 macrophages polarized by IL-13 treatment. (**A**) mRNA expression of components of NOX complexes in M2 macrophages was quantified with QPCR and normalized to the expression of β-actin mRNA. (**B**) The expression of ROS producing enzymes (NOX1, NOX2) and subunits (NCF1, NCF2, NCF4) of the NOX2 complex was determined with western blot. Densitometry was performed for each protein and normalized to actin. Densitometry values are noted below each band. Columns: mean, error bars: +SD, ^*^p<0.05 for comparison to the corresponding macrophages treated with vehicle. ^§^p<0.05 for comparison to C57L macrophages exposed to IL-13 by ANOVA with Tukey’s correction.

Overall, our findings suggested a relationship between the accumulation of alternatively activated (M2) macrophages and lung fibrosis in C57L and C57BL6/J mice. M2 macrophages are known to produce ROS and depend on NADPH oxidases for polarization [42]. As shown earlier, the expression of components of NOX2 was increased in irradiated C57L mouse lung at 15 weeks after IR. Thus, the expression of the NADPH oxidase isoforms *Nox1*, *Nox2*, and *Nox4*, were examined in M2 macrophages from each strain polarized by IL-13 with quantitative PCR and western blot. The expression of *Nox1* and *Nox2* were significantly increased in M2 polarized BMDM from C57L (*Nox1*: 3.9 ± 0.3, *Nox2*: 2.4± 0.1 fold) and C57BL6/J (*Nox1*: 1.7 ± 0.4, *Nox2*: 1.5 ± 0.03 fold) mice, but not in BMDM from C3H/HeN mice (Figure 6A). *Nox4* mRNA was not detected in both M0 and M2 BMDM derived from all strains. NOX1 and NOX2 protein were expressed at the highest levels in C57L BMDM, with intermediate expression in C57BL6/J BMDM and the lowest expression in C3H/HeN BMDM (Figure 6B).

The NOX2 subunits (*Ncf1, Ncf2, Ncf4*) are critical components of the active NOX2 complex, and as presented earlier, were expressed to a greater degree in the lungs of C57L mice compared to C57BL6/J and C3H/HeN as determined by transcriptional profiling of whole lung (Figure 4B). Therefore, the expression of these subunits was also examined also in M2 macrophages with quantitative PCR and western blot. The greatest degree of increased expression of each subunit was observed in M2 macrophages from C57L (Figure 6B). The increase of NOX2 subunits proteins was seen in M2 macrophages from C57BL6/J, but the level of mRNA didn’t present significant changes.

**Figure 7:**
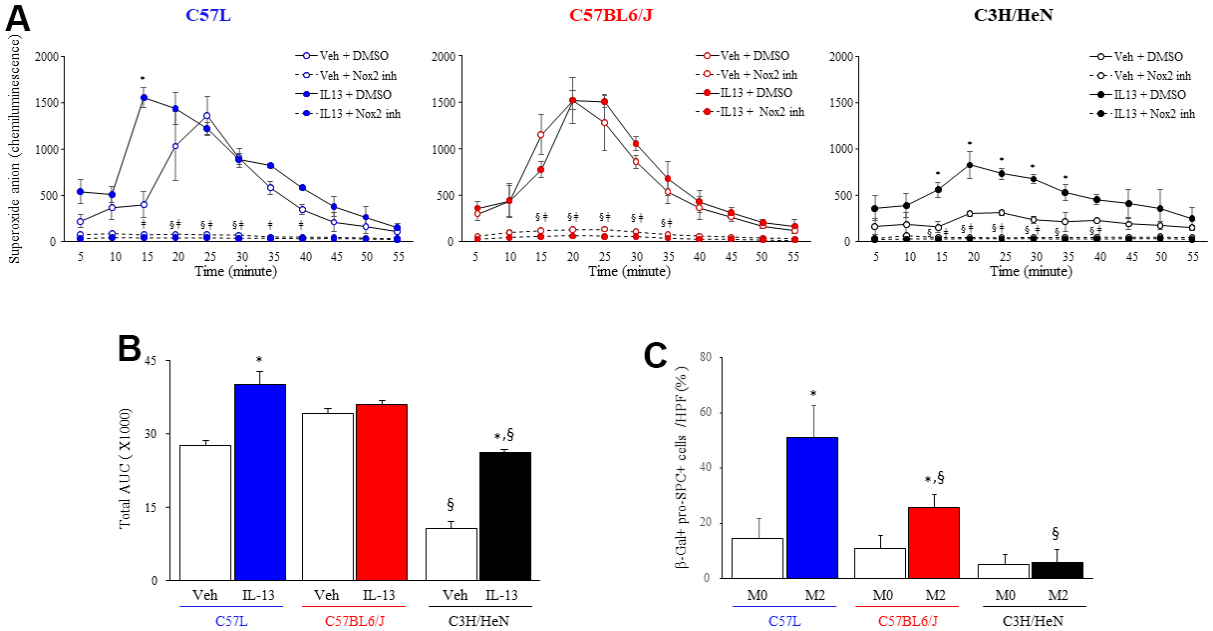
**NOX2-mediated macrophage superoxide production and senescence inducing capacity varies by strain.** (**A**) Superoxide production was measured by luminol-amplified chemiluminescence in M2 macrophages (IL-13 treated) stimulated with PMA (100 ng/ml) in the presence or absence of a NOX2 inhibitor, GSK2795039 (100 nM). Each symbol: mean, error bars: +SD, ^*^p<0.05 for comparison between macrophages with vehicle and IL-13, ^§^p<0.05 for comparison between macrophages with vehicle +/- GSK2795039, ^ǂ^p<0.05 for comparison between macrophages with L-13 +/- GSK2795039 by multiple T Test. (**B**) Area under the curve analysis for Superoxide production after IL-13 and PMA treatment. (**C**) Enriched primary AECII from the three strains of mice were seeded on transwell inserts (pore size: 0.4 μm) and cocultured for 3 days with syngeneic M2 macrophages polarized with IL13. AECII were fixed after 3 days, and senescence associated β-gal activity was assessed followed by confirmatory immunocytochemical localization of pro-surfactant C (pro-SPC). The percent of senescent AECII was scored. Columns: mean, error bars: +SD, ^*^p<0.05 for comparison to macrophages with vehicle. ^§^p<0.05 for comparison to C57L macrophages exposed to IL13 by ANOVA with Tukey’s correction.

These data suggested that macrophages from the three strains polarized with IL-13 may have differing capacities for superoxide production. ROS production in response to an NOX2 activator (Phorbol 12-myristate 13-acetate, PMA) was measured in IL-13 stimulated macrophages from each strain (Figure 7A, 7B). M2 macrophages from C57L and C57BL6/J mice exhibited rapid increases in ROS production in response to PMA that were similar to unpolarized macrophages (M0). Though M2 macrophages from C3H/HeN mice showed increased ROS production compared to M0 macrophages from C3H/HeN, the levels of ROS production from both M0 and M2 C3H/HeN macrophages were significantly lower than macrophages from other strains. ROS production in response to PMA was drastically inhibited by the treatment of specific NOX2 inhibitor, GSK2795039.

To determine if M2 macrophages were capable of inducing AECII senescence, and to explore if the observed differences in macrophage function had a relevance on the rate of AECII senescence, primary AECII cultures enriched from each strain were cocultured with their respective strain’s primary macrophage culture polarized with IL13 in a transwell system (Figure 7C). Coculture of IL-13 polarized M2 macrophages and AECII from the C57L strain resulted in a significant increase in AECII senescence compared to coculture with M0 macrophages. Similarly, AECII derived from C57BL6/J exhibited increased rates of senescence after exposure to M2 macrophages from C57BL6/J mice, but to a lesser degree than that observed in C57L co-culture. Co-culture of C3H/HeN M2 macrophages only minimally induced senescence in AECII enriched from C3H/HeN, consistent with the reduced production of ROS in stimulated IL-13 polarized C3H/HeN M2 macrophages observed previously. Taken together, these data suggest that macrophage function contributes to the differential susceptibilities to AECII senescence between the strains.

### Macrophages expressing NOX2 accumulate in fibrotic lungs after radiation

**Figure 8:**
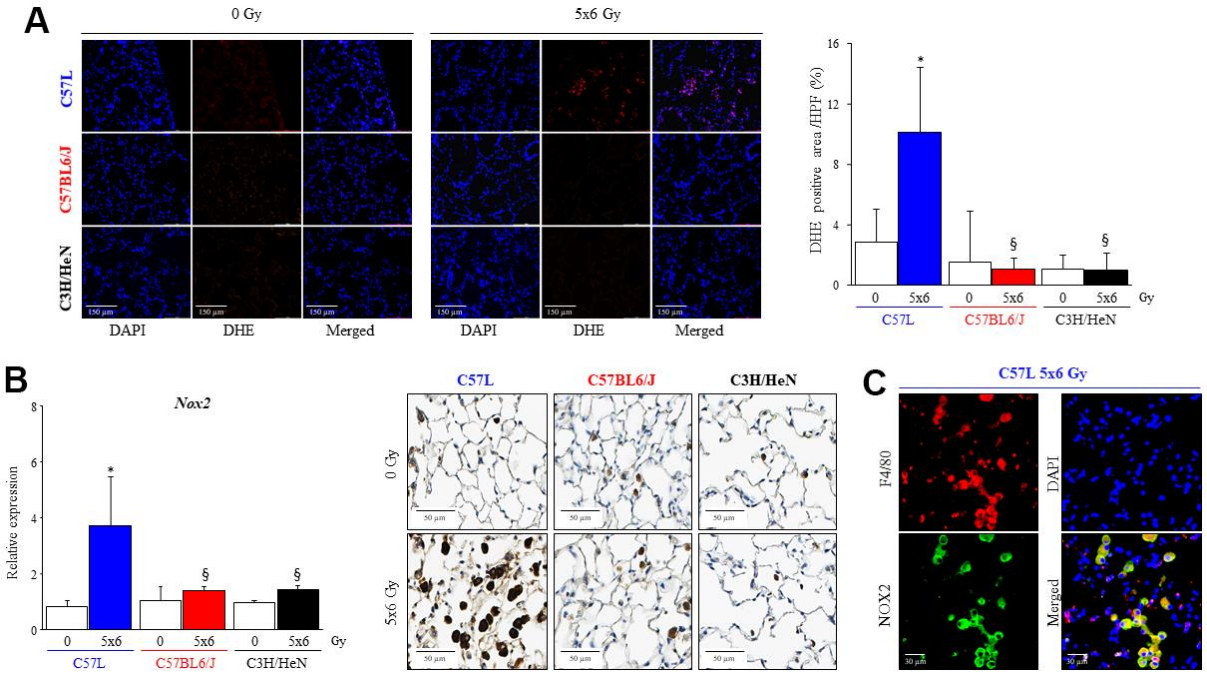
**Increased numbers of macrophages expressing NOX2 in fibrotic lungs.** C57L, C57BL6/J and C3H/HeN mice were exposed to 5 daily fractions of 6 Gy (5x6 Gy) of thoracic irradiation. (**A**) At 15 weeks after radiation, samples of frozen lung tissue were collected and dihydroethidium (DHE) oxidation was assessed. The level of cellular superoxide anion was quantified as the percentage of DHE-positive cell in each HPF (20x) region. (**B**) The expression of *NOX2* was examined by QPCR and immunohistochemical assays (NOX2: brown, Nucleus: blue) in mouse lungs from the three strains 15 weeks after irradiation. (**C**) NOX2 expressing cells in lung were identified as a macrophage with co-labeling for F4/80. Columns: mean, error bars: +SD, ^*^p<0.05 for comparison to each lung with 0 Gy. ^§^p<0.05 for comparison to C57L lungs exposed to 5x6 Gy by ANOVA with Tukey’s correction.

To determine if the differences in macrophage response to polarizing and activating stimuli seen *in vitro* correlated with *in vivo* findings, in the *in vivo* production of superoxide was assessed by dihydroethidium staining in frozen lung sections of each strain collected at 15 weeks after IR (Figure 8A). A significant increase in superoxide production was only observed in fibrotic foci in the lungs of C57L mice (0 Gy: 2.8 ± 2.2%, 5x6 Gy: 10.1 ± 4.3%). This finding corresponded to higher expression of *Nox2* mRNA after IR in C57L lung (0 Gy: 0.8 ± 0.2, 5x6 Gy: 3.7 ± 1.7 fold, Figure 8B). As assessed with immunohistochemistry, the presence of NOX2 expressing cells within the alveoli were observed in each strain, but a substantial increase in the number of these cells and intensity of staining was observed only in irradiated C57L mouse lung. These NOX2 expressing cells were confirmed to be alveolar macrophages with dual fluorescence staining (Figure 8B, 8C).

## DISCUSSION

In this study evaluating three mouse strains, we demonstrate that the strains with the greatest sensitivity (C57L) to radiation induced lung injury and fibrosis also exhibit the greatest rate of accumulation of senescent AECII, a pulmonary adult stem cell. Simultaneously, the fibrosis sensitive (C57L and C57Bl6/J), senescence accumulating mouse strains exhibit a greater accumulation of M2 polarized macrophages than a fibrosis resistant strain (C3H/HeN), with the greatest accumulation in perifibrotic areas. Further, strain-specific differences in macrophage polarization were described that are, in turn, associated with a strain dependent capacity of activated macrophages to induce premature epithelial senescence via NOX2 mediated superoxide production. This capacity of M2 macrophages from the most fibrosis sensitive strains to induce epithelial senescence *in vitro* via NOX2 aligns with the observed *in vivo* increase in accumulation of senescent AECII, alternatively activated macrophages, and increased NOX2 expression in the lungs of irradiated fibrosis sensitive mice.

A large body of work has described varying sensitivity to radiation, bleomycin, and fibrosis evoking stimuli [43] between strains of mice. After exposure to radiation, inbred mouse strains have been shown to have differences in the chronology of fibrosis [6, 44, 45], morbidity [6, 46], inflammation [18], immune polarization [18, 47], and cytokine expression [48–52]. However, the underlying mechanisms responsible for these differences in inflammatory responses and sensitivity to fibrosis have remained largely unexplored. Prior studies in the C57BL6 strain have documented the accumulation of senescent AECII and pulmonary parenchymal cells as a consequence of radiation [27], and have demonstrated that the clearance or prevention of senescence can prevent or reverse radiation fibrosis [8, 13, 14, 27]. However, despite this clear link between senescence and the progression of radiation fibrosis, differences in senescent cell accumulation in strains with varying sensitivity to fibrosis have not been explored. This study is the first to compare premature senescence among different mouse strains with varying sensitivity to radiation fibrosis and has identified that senescent cell accumulation occurs earlier in fibrosis sensitive strains with minimal accumulation of senescent cells in a fibrosis resistant strain. Based on this finding, further studies were then conducted to explore potential contributors to the variability in senescent AECII accumulation between strains.

The findings of accumulation of M2 macrophages in fibrotic progression are consistent with prior studies of radiation lung injury [8, 13, 14] and other fibrotic conditions [15–17]. In the context of strain variability, these findings are consistent with prior studies characterizing macrophage subpopulations over time in selected strains [18] and with a large body of work implicating M2 macrophages in fibrotic progression from many causes [17, 53–57]. In a prior study that evaluated gene expression in C57L and C57BL6/J mouse lung 24 hours after fibrosis evoking doses of radiation, *Retnla* was identified as a gene differentially expressed after lethal exposures [46]. The expression of resistin-like alpha (RELM-α), the protein encoded by *Retnla* and a marker of M2 macrophages, was found to be increased in C57L, and to a lesser degree C57Bl/6J mice, after radiation [46]. These data are consistent with our observation that C57L mouse lungs exhibit a greater degree of M2 macrophage accumulation relative to C57BL6/J mouse lungs after irradiation.

Another key finding from this study is the observation that macrophages derived from strains with varying sensitivity to fibrosis and varying rates of senescent cell accumulation exhibit intrinsic differences in response to M2 polarizing stimuli, and that these M2 polarized macrophages are capable of causing AECII senescence through secreted factors. M2 polarized macrophages were previously described as the dominant immune cells that accumulate in irradiated lung during fibrotic progression [8]. Although macrophages have been described to play an important role in wound repair [58–61] and lung homeostasis [11, 62, 63], the crosstalk between injured pulmonary epithelia and macrophages after injury are incompletely understood. Senescent irradiated AECII have been shown to induce secondary senescence in bystander cells [27] and to have the capacity to polarize macrophages toward to M2 phenotype through elaboration of SASP molecules [13, 14]. However, the capability of M2 polarized macrophages to amplify this process by inducing senescence in AECII provides the first evidence of a positive feedback loop that may further drive chronic fibrotic progression.

Recently, it has been described that molecules secreted by senescent AECII, such as IL-13 and growth differentiation factor 5 (GDF15) [14, 25, 26, 64] are capable of driving macrophage polarization to an M2 phenotype. However, until now, the impact of M2 polarization on AECII senescence was unexplored. In this study, we identified that M2 macrophage polarization can contribute to AECII senescence, potentially leading to a positive feedback loop that furthers pulmonary injury.

In the current study, transcriptional profiling of irradiated lung identified an enrichment in the “ROS and NO production in phagocytes” gene set in the fibrosis sensitive C57L strain, with increased expression of multiple subunits of NADPH Oxidase 2 within this gene set. Chronic oxidative stress as a result of ongoing superoxide production by NADPH Oxidases has been implicated as a contributor to fibrosis in irradiated lung, with NOX inhibition sufficient to prevent both AECII senescence and RIPF [27]. Based on the importance of both senescence, M2 macrophages, and NADPH Oxidases in fibrotic progression, we focused on the capacity of M2 polarized macrophages from the three strains of mice to produce superoxide. NOX2 is highly expressed in macrophages and inflammatory cells and plays a key role in the host response to pathogens [65–67]. NOX2 has been described to play a pathologic, protective, or modulatory role [68–70] in lung injury and inflammation, reflecting that the cell of expression and context may be an important contributing factor. In the context of macrophages, reactive oxygen species produced by NOX1 and NOX2 play an important role in the differentiation of monocytes to macrophages and in the polarization of macrophages to an M2 phenotype [41]. Loss of NOX1 and NOX2 has been described to have no effect on macrophage inflammatory response after exposure to M1 polarizing stimuli [41]. The finding in this study that superoxide production in M2 macrophages and was NOX2 dependent and observed to the greatest degree in macrophages derived from fibrosis sensitive strains provides the first evidence that macrophages from these three strains of mice respond differently to polarizing stimuli and further support that M2 macrophage function and strain-based differences are partially dependent on NOX2. Prior studies of immune function between mouse strains have examined free radical production of neutrophils derived from different strains of mice, but neutrophils are a minor component of the inflammatory cell infiltrate beyond the acute phase [71, 72]. Although NOX2 has not been previously implicated in the context of lung injury, a comparative proteomic analysis of irradiated C3H/HeJ and C57BL/6J lung tissue identified several anti-oxidant/redox related proteins were demonstrated to be down regulated in the fibrosis sensitive strain [73], however in whole lung protein lysates, NADPH oxidases were not one of the identified proteins with differential expression. Similar to our findings, oxidative stress was noted to be higher in the lungs of irradiated C57BL/6J mice compared to C3H/HeJ mouse lung [73].

It will be important to further explore these findings in additional strains of mice with varying responses of macrophages to fibrotic stimuli as mouse strain variation has been hypothesized to correlate to human natural immune variation [74]. Future work is underway to explore mechanisms of strain dependent variation in macrophage response to polarizing stimuli. Further, it is unclear if the differences observed in inbred mouse strains will translate to individual variation in sensitivity to lung injury in human patients exposed to thoracic radiotherapy, though these are the focus of future studies. This study has not addressed the impact of senescent cell clearance through immune surveillance, which may also contribute to a lack of accumulation of senescent cells over time [75, 76]. As M1 macrophages may contribute to senescent cell clearance, this may impact the rate of senescent cell accumulation in the C3H/HeN strain in particular, as a significant increases M1 macrophages were observed in irradiated C3H/HeN lungs at 32 weeks after IR. Interestingly, M0 macrophages from C3H/HeN produced CCL17 and CCL22 abundantly in response to IL13 treatment. CCL17 and CCL22 are well known as a cytokine attracts and activates CCR4+ immune cells such as cytotoxic and helper T cell, NK cells and regulatory T cells which are involved in the clearance of senescent cells [77–79]. Future studies will attempt to elucidate senescent cell clearance in strains with varying sensitivity to RIPF.

There are some additional limitations of this study, including an inability to directly implicate macrophages in senescence *in vivo* with clearance of a specific macrophage polarization subtype, as macrophage depletion techniques are generally non-specific to polarization status. Further, the macrophages implicated in the induction of senescence share markers of both M2a and M2c polarization. Thus, they have not been further subclassified beyond M2 in these studies. Studies to specifically inhibit M2a and M2c may be required *in vivo* to further clarify this issue. Additionally, the studies herein were conducted with female mice using a single radiation fractionation regimen. It is not known if the effects observed here would be similar to those observed in male mice, as strain dependent variation in radiation response and macrophage function has been reported [6, 19–22]. Further, it is not clear if similar results would be obtained with different total radiation doses or single fraction delivery. As pneumocyte senescence has been shown to be radiation dose dependent [27] and radiation lung injury is known to be dose radiation dependent [80], it is reasonable to hypothesize that radiation dose may also modulate macrophage infiltration and function and impact these findings. Future studies should systematically evaluate whether these variables also can impact macrophage function and injury.

## CONCLUSIONS

In this study, variation in the accumulation of senescent cells across strains with varying sensitivity to fibrosis has been established. Further, strain variation in macrophage response to polarizing stimuli and capacity to produce superoxide and induce senescence in epithelial cells is described. Together, these data highlight the importance of macrophage-epithelial interactions in the context of lung fibrosis and identify NOX2 as a possible therapeutic target in radiation lung injury.

## Supplementary Material

Supplementary Figures

Supplementary Tables
