# Supplementary material for: Natural variation in macrophage polarization and function impact pneumocyte senescence and susceptibility to fibrosis
Source: Aging (Albany NY). 2022 Sep 28;14(19):7692–717. doi: 10.18632/aging.204309 (PMC9596223; doi:10.18632/aging.204309)
Supplement: Supplementary Figures [file aging-14-204309-s001.pdf]

SUPPLEMENTARY FIGURES

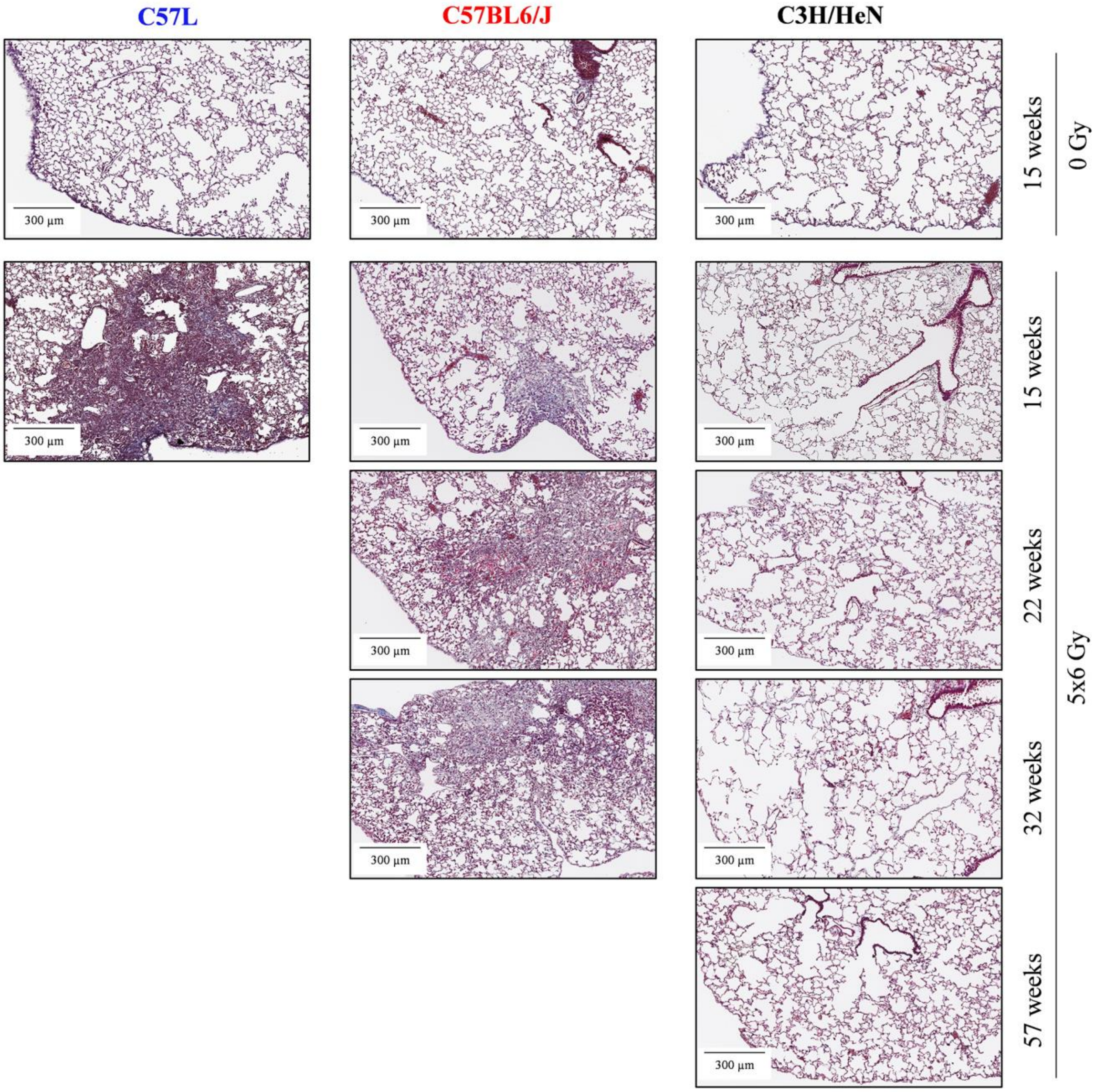

Supplementary Figure 1. Fibrotic regions of lung after thoracic radiation. C57L, C57BL6/J and C3H/HeN mice were exposed to 5 daily fractions of 6 Gy (5x6 Gy) of thoracic irradiation. To determine fibrotic regions, sections of lung from each strain collected at the time points were subjected to Masson Trichrome staining.

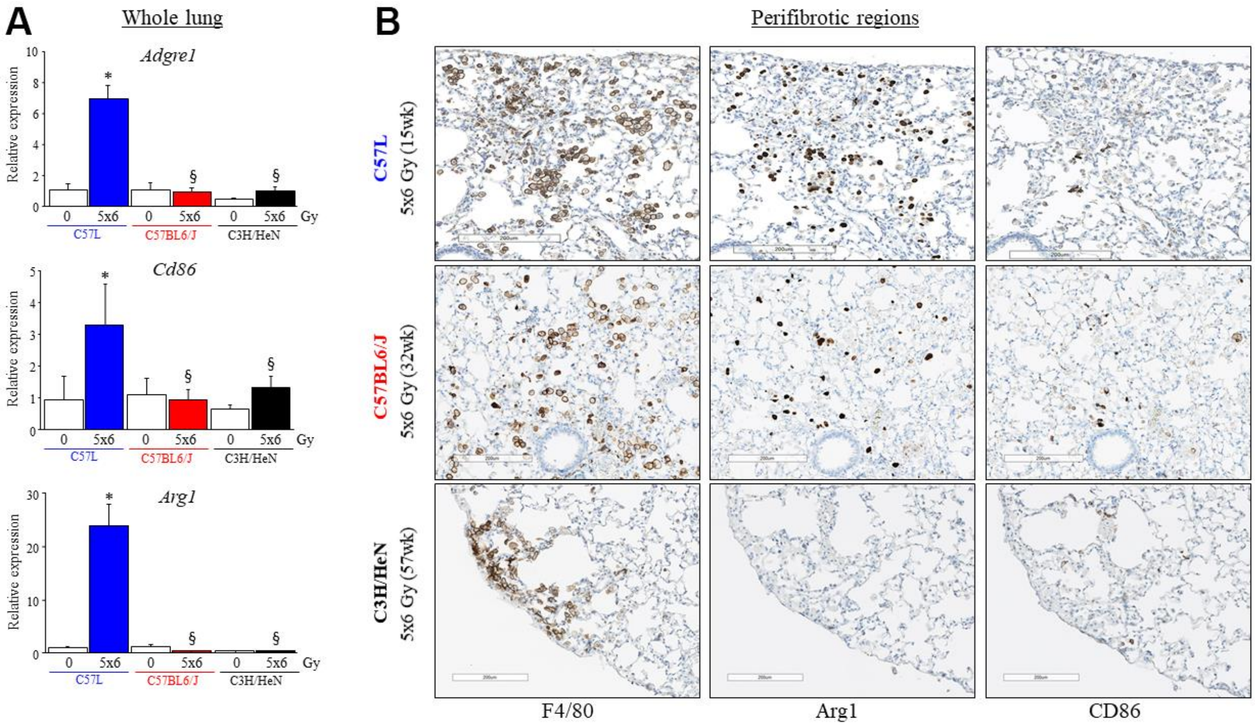

Supplementary Figure 2. Determination of different types of macrophages in mouse lungs. C57L, C57BL6/J and C3H/HeN mice were exposed to 5 daily fractions of 6 Gy (5x6 Gy) of thoracic irradiation. At 15, 32 and 57 weeks after irradiation, lung tissues were collected for further assays. (**A**) RNA was isolated from lung tissues and analyzed with QPCR to compare the expression of M1, and M2 macrophage markers (total: *Adgre1*, M1: *Cd86*, M2: *Arg1*). Relative changes of each marker were normalized to *β-actin*. (**B**) The expression of F4/80, CD86 and Arginase-1 (brown) was detected with immunohistochemistry with hematoxylin counterstaining (nuclei, blue) in perifibrotic regions of lung. Representative images are presented. Columns: mean, error bars: +SD, $^*p<0.05$ for comparison to lung with 0 Gy. $^§p<0.05$ for comparison to C57L lung exposed to 5x6 Gy by ANOVA with Tukey's correction.

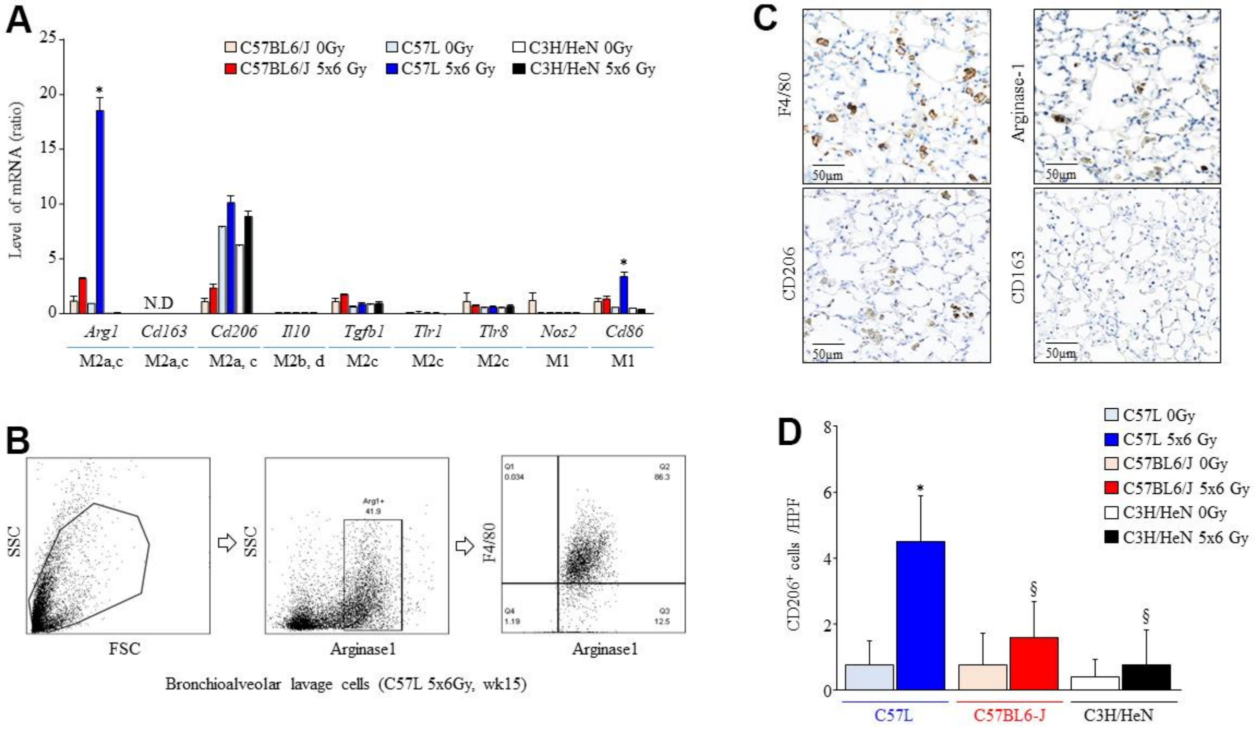

Supplementary Figure 3. Characterization of macrophages in bronchioalveolar lavage fluid from three strains. C57L, C57BL6/J and C3H/HeN mice were exposed to 5 daily fractions of 6 Gy (5x6 Gy) of thoracic irradiation. At 15 weeks after irradiation, mononuclear cells were collected from bronchioalveolar lavage fluid (n≥5 mice per condition). (**A**) RNA was isolated from bronchioalveolar cells and analyzed with QPCR to compare the expression of M1, and M2 macrophage markers. Relative changes of each marker were normalized to *β-actin*. (**B**) Expression of F-4/80 and Arginase-1 was assessed by flow cytometric assay in bronchioalveolar cells. (**C**) The expression of F4/80, Arginase-1 and CD206 (brown) was detected with immunohistochemistry with hematoxylin counterstaining (nuclei, blue) in lung. Representative images are presented. (**D**) The numbers of CD206+ cells were scored in whole lung. Columns: mean, error bars: +SD, $^{*}p<0.05$ for comparison to lung with 0 Gy. $^{\S}p<0.05$ for comparison to C57L lung exposed to 5x6 Gy by ANOVA with Tukey's correction.

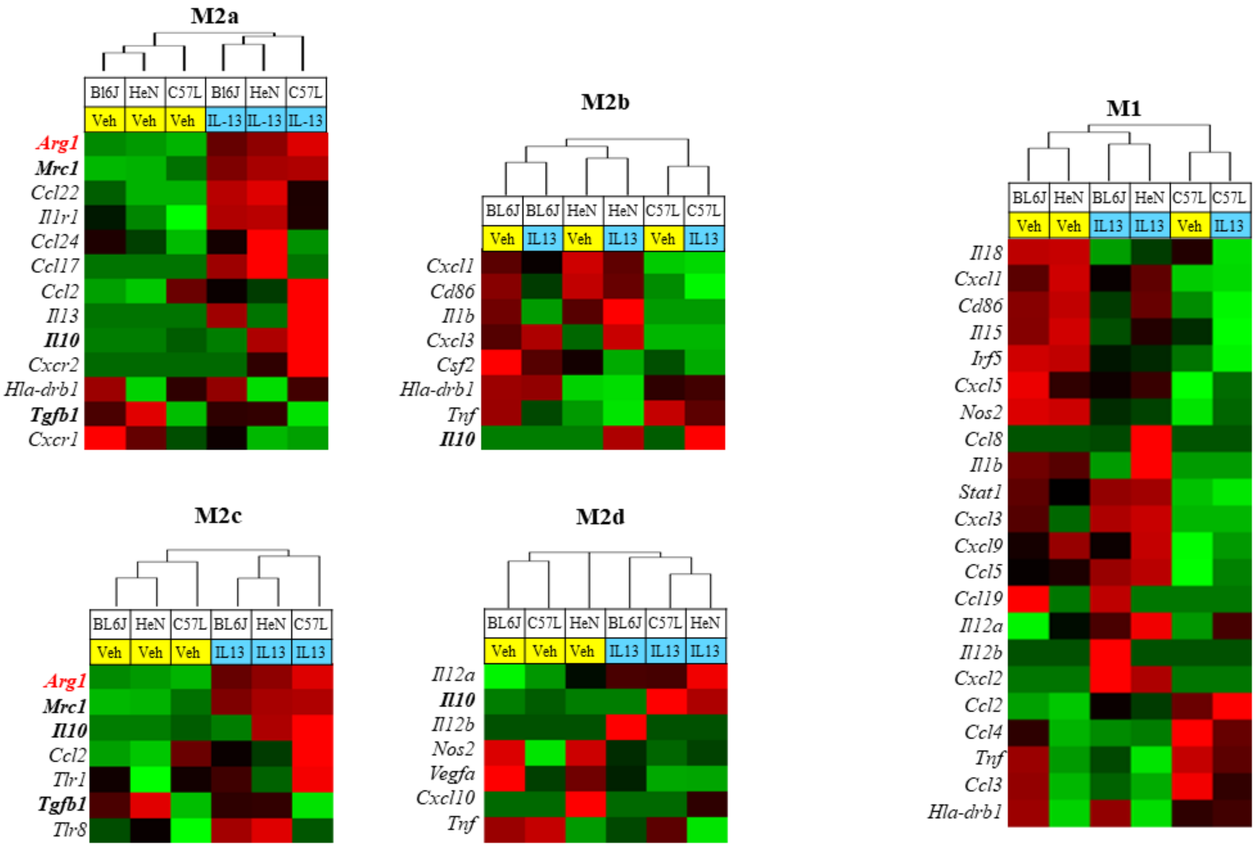

Supplementary Figure 4. Characterization of macrophage phenotype across mouse strains after exposure to IL-13. Bone marrow derived macrophages from each strain were polarized with vehicle (PBS) or IL-13 (10 ng/ml). After 3 days of exposure, total RNA was isolated. The expression of genes related to M1 and M2 polarization was evaluated in macrophages treated with vehicle or IL-13 using the NanoString nCounter Gene Expression Assay and a custom code set. Unsupervised hierarchical clustering of genes relating to each polarization subtype was performed.
