# Supplementary material for: Natural variation in macrophage polarization and function impact pneumocyte senescence and susceptibility to fibrosis
Source: Aging (Albany NY). 2022 Sep 28;14(19):7692–717. doi: 10.18632/aging.204309 (PMC9596223; doi:10.18632/aging.204309)
Supplement: Supplementary Tables [file aging-14-204309-s002.pdf]

SUPPLEMENTARY TABLES

Supplementary Table 1. NanoString code set.

Gene name (HUGO)	Accession	Position	Target sequence
ARG1	NM_000045.3	674-773	TCAATGACTGAAGTGGACAGACTAGGAATTGGCAAGGTGATGGAAGAAACACT<br>CAGCTATCTACTAGGAAGAAAGAAAAGGCCAATTCATCTAAGTTTTG
CCL17	NM_002987.2	230-329	GCCTGGAGTACTTCAAGGGAGCCATTCCCCTTAGAAAGCTGAAGACGTGGTACC<br>AGACATCTGAGGACTGCTCCAGGGATGCCATCGTTTTTGTAACTGT
CCL2	NM_002982.3	124-223	CATTCCCCAAGGGCTCGCTCAGCCAGATGCAATCAATGCCCCAGTCACCTGCTGT<br>TATAACTTCACCAATAGGAAGATCTCAGTGCAGAGGCTCGCGAGC
CCL22	NM_002990.3	798-897	CTCGCCCAAGCAGCTGGTAATTCCATTTCATGTATTAGATGTCCCCTGGCCCTCT<br>GTCCCCTCTTAATAACCCTAGTCACAGTCTCCGCAGATTCTTGGG
CCL24	NM_002991.2	19-118	ATAGTAACCAGCCTTCTGTTCCTTGGTGTCTGTGCCCACCACATCATCCCTACGG<br>GCTCTGTGGTCATCCCCTCTCCCTGCTGCATGTTCTTTGTTTCCA
CXCR1	NM_000634.2	1951-2050	GCAGCCACCAGTCCATTGGGCAGGCAGATGTTCCTAATAAAGCTTCTGTTCCGTG<br>CTTGTCCCTGTGGAAGTATCTTGGTTGTGACAGAGTCAAGGGTGT
CXCR2	NM_001557.2	2056-2155	AGGAGAAACTGGAACTCTCGAGCGTTGCTGGGGGGGATTGTAAAATGGTGTGAC<br>CACTGCAGAAGACAGTATGGCAGCTTTCCTCAAAACTTCAGACATA
HLA-DRB1	NM_002124.3	748-847	AGCACGGTCTGAATCTGCACAGAGCAAGATGCTGAGTGGAGTCGGGGGCTTTGT<br>GCTGGGCCTGCTCTTCCTTGGGGCCGGGCTGTTCATCTACTTCAGG
IL10	NM_000572.2	231-330	AAGGATCAGCTGGACAACTTGTTGTTAAAGGAGTCCTTGCTGGAGGACTTTAAG<br>GGTTACCTGGGTTGCCAAGCCTTGTCTGAGATGATCCAGTTTTACC
IL13	NM_002188.2	517-616	TTTCTTTCTGATGTCAAAAATGTCTTGGGTAGGCGGGAAGGAGGGTTAGGGAGG<br>GGTAAAATTCCTTAGCTTAGACCTCAGCCTGTGCTGCCCGTCTTCA
IL1R1	NM_000877.3	583-682	TGCTAAGGTGGAGGATTCAGGACATTACTATTGCGTGGTAAGAAATTCATCTTA<br>CTGCCTCAGAATTAAAATAAGTGCAAAATTTGTGGAGAATGAGCCT
MRC1	NM_002438.3	941-1040	TGACCTCAGGACTCTGGATTGGACTTAACAGTCTGAGCTTCAACAGCGGTTGGC<br>AGTGGAGTGACCGCAGTCCTTTCCGATATTTGAACTGGTTACCAGG
TGFB1	NM_000660.3	1261-1360	TATATGTTCTTCAACACATCAGAGCTCCGAGAAGCGGTACCTGAACCCGTGTTGC<br>TCTCCCGGGCAGAGCTGCGTCTGCTGAGGCTCAAGTTAAAAGTGG
**Internal reference gene**	**Accession**	**Position**	**Target sequence**
CLTC	NM_004859.2	CLTC	GGGTATCAACCCAGCAAACATTGGCTTCAGTACCCTGACTATGGAGTCTGACAA<br>ATTCATCTGCATTAGAGAAAAAGTAGGAGAGCAGGCCCAGGTGGTA
GAPDH	NM_002046.3	GAPDH	ACTTCAACAGCGACACCCACTCCTCCACCTTTGACGCTGGGGCTGGCATTGCCCT<br>CAACGACCACTTTGTCAAGCTCATTTCCTGGTATGACAACGAATT
GUSB	NM_000181.1	GUSB	CGGTCGTGATGTGGTCTGTGGCCAACGAGCCTGCGTCCCACCTAGAATCTGCTG<br>GCTACTACTTGAAGATGGTGATCGCTCACACCAAATCCTTGGACCC
HPRT1	NM_000194.1	HPRT1	TGTGATGAAGGAGATGGGAGGCCATCACATTGTAGCCCTCTGTGTGCTCAAGGG<br>GGGCTATAAATTCTTTGCTGACCTGCTGGATTACATCAAAGCACTG
PGK1	NM_000291.2	PGK1	GCAAGAAGTATGCTGAGGCTGTCACTCGGGCTAAGCAGATTGTGTGGAATGGTC<br>CTGTGGGGGTATTTGAATGGGAAGCTTTTGCCCGGGGAACCAAAGC
TUBB	NM_178014.2	TUBB	TTCTAAGTATGTCCATTTCCCATCTCAGCTTCAAGGGAGGTGTCAGCAGTATTAT<br>CTCCACTTTCAATCTCCCTCCAAGCTCTACTCTGGAGGAGTCTGT

Supplementary Table 2. Primers and probes set for TaqMan gene expression assay.

Gene symbol	Target name	Assay ID
Adgre1	F4/80	Mm00802529_m1
Arg1	Arginase-1	Mm00475988_m1
Ccl2	CCL2	Mm00441242_m1
Ccl17	CCL17	Mm01244826_g1
Cd86	CD86	Mm00444540_m1
Cybb	NOX2	Mm01287743_m1
Il10	IL10	Mm99999062_m1
Mrc1	MRC1	Mm01329359_m1
Ncf1	NCF1	Mm00447921_m1
Ncf2	NCF2	Mm00726636_s1
Ncf4	NCF4	Mm00476300_m1
Nox1	NOX1	Mm00549170_m1
